# Notes on Medical Matters and Medical Men in Paris

**Published:** 1847-03

**Authors:** David W. Yandell

**Affiliations:** Louisville, Ky.; Paris


Art. V.—*Notes on Medical Matters and Medical Men in Paris.* By David W. Yandell, M.D., of Louisville, Ky.

In my last letter I alluded to the lecture of Professor Dumas, introductory to the winter course of instruction in the Ecole de Médicine, as a discourse of great beauty and eloquence. So much of it as related to the life and services of his eminent friend and associate, Bèrard, would probably be uninteresting to the readers of the Journal; but the conclusion of it presents views of great and general scientific interest. I propose to give a translation of the conclusion of the lecture.

In southern seas, remarked the distinguished professor, there are islands which slowly elevate themselves from the bottom of the ocean. Formed by polypi, these isles are visited by certain tribes of plants, and become the seat of an active vegetation; the remains of these plants collect upon the surface and form a soil, in which other races of vegetables spring up; animals and men seize upon them, at length, and the germ of a new empire appears upon the earth. It is curious to observe, that these corals direct incessantly and with unwearied energy their labors from within outwards, by which the land is made to encroach continually upon the sea. What peculiarity in their organization, what law governing their countless family, leads to results so favorable to conquest, that their works gain unceasingly upon the empire of Neptune? Do not lose yourselves in vain conjectures. We shall see that the sublime result is brought about by an agency simple in its nature and easily understood.

These polypi have need of calcareous matter to construct their dwellings; they find it in solution in the waters of the sea, and separate it in order that it may traverse their earthy tissue. This calcareous matter is rare within those immense bodies which elevate themselves from the sea, while it abounds without; and here, according to M. Forchammer, lies the whole secret of this providential form, of this eccentric tendency of their labors. Here is the part that mineral matter in general, that calcareous matter in this particular case, plays in the development of organized beings. Is not the spectacle which nature offers, in the sublime simplicity of her means, full of grandeur? Rain-water loaded with the carbonic acid of the air falls upon our limestone hills; it then becomes charged with carbonate of lime, reaches the Seine, is borne to the ocean, where the waves carry it away, and, ere long seized by microscopic animals, it adds an imperceptible stone to the formation of the new empires which are preparing for future humanity.

The phosphate of lime constitutes the base of the skeleton of all the superior animals, and is found both in the tissues and liquids of their economy. Analysis detects it in the inferior animals, and in the plants themselves. The phosphorus which this salt contains is found in milk and analogous liquors, and figures in its turn in a mysterious manner in the composition of the cerebral and nervous substance. But phosphorus and the phosphate of lime are so rare in nature, that, struck with the difficulty experienced by the earth in furnishing it to plants, an illustrious chemist exclaimed, "Rome fell when Sicily, drained of her phosphate of lime, was no longer able to furnish the grain necessary for her immense population." It is necessary that this phosphate of lime shall return to the earth, and to insure this, what simple and ingenious means does nature employ! Collected from the soil by the plants, these phosphates pass into the herbivorous animals, and from them into the systems of the carnivorous tribe, where it is concentrated. From this moment, everything tends to disperse them. If the animal dies, a fly deposits its eggs in the flanks of the body, millions of larvæ are generated, which feed upon the flesh and blood, pursue the course of their metamorphoses, and soon taking wing, in their turn bear away and scatter in all directions the phosphates which they have assimilated.

It is not without an end that nature has ordained, that the putrid remains, naturally a prey to the insects which devour them, should be, to animals higher in the scale of organization, objects of profound repugnance. Repulsed by the aspect and by the odor of these infectious bodies, they witness afar off the part that is here accomplished.

If the insects everywhere disseminate the phosphates contained in the flesh of dead bodies, hyenas, jackals and dogs devour the bones, and in their turn play the same part. But this is not all. Left to themselves upon the soil, the bones are gradually divided, and at length disappear. What new force intervenes to separate their elements?

According to my view of the matter, it is water, not pure, however—for in pure water phosphate of lime is insoluble—but water charged with carbonic acid—rain and spring water, that which everywhere moistens the soil. By the aid of this carbonic acid, phosphate of lime is dissolved, the bones crumble, and the last vestiges of animal life disappear. As you already know, it is this carbonic acid dissolved by the rains, entering into plants and decomposed under the influence of the solar rays, which forms their principal nourishment. Admirable mechanism, by which, according as the carbonic acid is consumed in the leaves of plants, phosphate of lime again becomes insoluble and is deposited in the vegetable tissues! What part is here played? One which is indispensable; for it is by it that all azotized matters resist the action of water, which tends to dissolve, to swell, and to separate them. It gives to our tissues their stability, renders our bones firm and unyielding, while it protects in the same manner by its presence the tissues of the plants.

Perhaps you may conceive that at the moment at which a molecule of carbonic acid is decomposed in a leaf—that at the moment in which the phosphate of lime held by it in solution becomes free—it is then that, seizing the albumen of the plant, those cloudy flocculi are produced which are the first origin of the cellules which one sees appearing every moment.

Return now and follow the air which penetrates the cells of your lungs; which is dissolved in your blood, there to burn the carbon which it contains, and reproduce the carbonic acid which plants constantly decompose. The venous blood will now contain carbonic acid, which is capable of rendering soluble the phosphate of lime. The venous blood will tend then, as we have seen rain-water does, to dissolve our bones, to swell and break down all our tissues, and all the cellules which compose them. Under its influence, the animal matter is carried away to be burned, in order to generate that heat which is necessary for the system, and the phosphate of lime dissolved, in order to be evacuated by the urinary secretions.

Thus a drop of water charged with carbonic acid, and dissolving the phosphate of lime, struck by the rays of the sun—behold the life which commences! In a drop of venous blood saturated with carbonic acid, and wasting our tissues, from which it has removed their phosphate of lime—behold the life which is finished! In the plant, it is a cellule which is organized; in the animal, a cellule which is dissolved: in the former, carbonic acid is decomposed; in the latter, carbonic acid is reproduced: in one, phosphate of lime becomes insoluble; in the other, it is redissolved. And these feeble efforts people the earth and the seas with numberless beings, who embellish or animate its surface, who feel, who think, and ever bear testimony to the omnipotence of nature.

Shall I show you now sulphur traveling from one kingdom to another, rising from the seas with vapor into the atmosphere, returning from the air to the soil, in plants, in animals, and redescending by the rivers, which again transport it to the sea? How simple, and yet how efficacious and sure is the mechanism of all these mutations! The sea contains the sulphates which nourish the mollusca. The humors which they secrete, greedy of oxygen, convert these sulphates into sulphurets. The water of the sea liberates sulphuretted hydrogen; the air carries it rapidly away until it encounters the remains of certain plants, whose pores by a mysterious power oblige it to burn itself, and thus produce sulphuric acid. The sulphates from this moment are reformed. This sulphuretted hydrogen, which is discharged from animal and vegetable matter in a state of decomposition—from sewers, from the subsoil of our streets—which tarnishes all our pictures—this sulphuretted hydrogen is one of the most indispensable terms of one of those grand equations by which the balance of nature is preserved. Two millions of kilogrammes of sulphur are required annually by the population of France, and not less than ten millions of kilogrammes will represent the mass contained in the whole of the organized beings that inhabit this portion of the globe.

The sulphates which the soil receives yield their sulphur to the plants, which in their turn furnish it to the animals; and in this way the earth would soon be exhausted of this element so necessary to the being of plants and animals, if the sea did not continually supply it. Admirable laws of nature, which incessantly array against each other the two kingdoms—which, in permitting the multiplication of animals, augment at the same time the growth of plants destined to become their food —which order, that as rapidly as vegetation increases, the air that it purifies and the resources that it develops, may be in their turn an exciting cause of the production of animals!

Shall we present to your attention the singular contrast between potash and soda, the former of which concentrates itself especially in plants, the latter particularly in animals? Our excretions carry off the potash, and return it to the earth, to the great benefit of vegetation; the water that we drink always contains the marine salt; our aliments abound in it, and thus is preserved, notwithstanding the continual losses, that which is required by our blood. This potash is soluble; it is washed from the soil into the rivers; from thence it is borne to the sea; and to what artifices has not agriculture resorted in order to restore it to the impoverished lands? It is potash which is contained in the ashes that are spread upon our fields; it is that which gives in an important degree richness to the manures of our farms; it is that which the lime thrown upon the soil gradually displaces in the alkaline silicates contained in all the argillaceous earths. While soda abounds in the waters of the sea, potash is found there also; and the marine plants, equally sensible in this respect with the terrestrial plants, condense in their tissues the salts of potash, and retain in the smallest possible proportions the marine salt, which traverses their tissues in such enormous quantities.

Is it not by returning to the soil exhausted of its potash this alkali, which his wines continually hold in the form of cream of tartar, that the agriculturist of the environs of Montpelier manures with such success his vines by means of sea-weeds? Is it not also in a great measure owing to the salts of potash, existing in these sea-weeds, so abundant on the shores of the ocean, which, thrown upon the fields of the coast, insures them such invariable fertility?

Having been proved that this sea-water is so rich in salts of potash, why should it not itself become the base of an excellent manure? I should rejoice if this thought might be confirmed. I would like to see this water of the sea, into which all the residues of life are drifted and there mingled together, separated into two parts obedient to the hand of man—yielding him, in the crystalizable salts which he has abandoned, soda, a true aliment for him and the animals that are associated with his destiny; leaving, in the salts which do not crystalize, potash, an element indispensable to the vigor of the plants which he cultivates.

These great examples suffice. It is unnecessary to describe how iron is concentrated in the leaves of plants, in the blood of animals; how the fluoride of calcium follows the course of phosphate of lime, and associates itself with it in the enamel of our teeth; how silex enriches the grasses, and exists pure in the blood of animals; how, on the contrary, it takes the place of their soft tissues in so many fossils.

The police of Paris watches over the restaurants with a keen eye, and all its scrutiny is demanded by the public health. Animals now must all be killed within the *Barriérs*, and so strict is the watch that it is nearly impossible to introduce flesh forbidden by law on account of its unwholesome character or its being that of interdicted animals. Some years ago large quantities of horse flesh were constantly sold at the cheap dinner houses for beef. In a short time, two thousand kilogrammes were intercepted by the police as it was about to enter the city. At another time, the officers seized upon nine hundred kilogrammes of horse oil, which was to have been used by the lower restaurants in sauces instead of butter. A lady of wealth in the Place Vendôme used a *milk bath* for her health, and the milk was afterwards sold to the keeper of a coffee house in the Rue St. Honoré, and served out to his customers in their coffee. A baker was convicted of having conveyed the urine from the *lieux d'aisance* into his establishment to save the expense of water. Where such atrocities are committed, it is needful that there should be a vigilant police.

The celebrated Italian physician Tommasini died on the 26th of November last, at Parma—where he was born in 1769—of acute bronchitis, in the 77th year of his age.

On the dissecting table of one of my acquaintances at the *Ecole Pratique* is a young subject, in the head of which an unusual distribution of the temporal artery exists. The common carotid divides below the level of the cricoid cartilage. The temporal artery comes off along with the occipital by a common trunk of an inch and a half in length; the temporal proceeds from its anterior side, runs along the inner surface of the posterior belly of the digastric muscle, and external to the styloid process, being concealed in this position by a portion of the parotid gland. It then proceeds upward and outward in contact with the forepart of the root of the mastoid process; afterwards it forms an arch, the concavity of which looks forward to the external ear, behind which it curves to gain about two inches above the zygoma, its usual situation, when it divides into its principal branches. The internal maxillary furnishes a small posterior auricular artery, and also a little higher another branch is seen in the normal position of the trunk of the temporal artery, which sends off the anterior auricular and transversalis facial arteries as well as smaller twigs to the neighboring parts.

Dr. Jaesche, of Minsk, has published a method of treating *prolapsus ani* without the knife, which possesses in his opinion many advantages over the usual operation. The treatment consists in applying to the anus, or even introducing a line or a line and a half into the rectum, a piece of lint dipped in a weak solution of nitric or sulphuric acid, though the former is preferable, being less irritating and painful. The pain which succeeds this cauterization yields in a short time to the application of lint saturated with olive oil, and the excoriations recover in the space of a few days. One or two cauterizations have generally been sufficient in Dr. J.'s hands for the complete recovery of the numerous patients whom he has treated for this affection.

M. Guillon presented, at a late meeting of the Academy of Medicine, an individual on whom he had successfully operated for a fibrous and very hard stricture of the urethra. Before the operation, he was unable to introduce into the canal the smallest sound; afterwards, he could introduce one of considerable calibre.

The following interesting case, published in a German journal, occurred in the practice of Professor Naegelé, jr., of Hiedelberg, son of the celebrated accoucheur of that name: A woman, aged thirty-two years, a *primipara*, arrived at the term of her pregnancy; at the moment when labor commenced, auscultation was practised, and there was distinctly heard in the left hypogastric region the two sounds of the fœtal heart and a simple *bruit de souffle* of the cord, and in the right hypogastric region quite as distinctly other two beats. Two operators applied the stethoscope, one to the right the other to the left side of the woman, and found the beats of the heart of the fœtus and of the cord more frequent on the right than the left. At a later period, when the right fœtus made more movements, the pulsations were forty for the heart, and thirty-six for the cord, which would make a difference of sixteen pulsations in a minute. After the delivery of the first child by the forceps, auscultation was again performed, and pulsations of the fœtal heart, both in the right umbilical region and left hypocondria, were clearly heard. The head of the second fœtus presented itself at the orifice in the second position; the noise of the heart heard in the right side appertained to this fœtus, which was delivered like the other by the forceps. Pulsations of a single heart were now heard, which were produced by the third fœtus, which came after a time into the world without the aid of art. Fifty-seven hours were occupied by this labor.

The diagnosis of the presence of more than one fœtus in the uterus was founded upon the two double sounds of the fœtal hearts. It was not until after the birth of the first child, when other distinct double sounds were heard, that it was supposed to be a pregnancy with three children. The noise of the heart of the third child had not been heard in the commencement of labor, probably because it was placed behind the others, towards the spine of the mother. The uterine *bruit de souffle* was neither more loud nor more extensive than in ordinary pregnancy.

In the *Bulletin de Therapeutique* there is a case of chorea reported, which was cured in the wards of M. Trousseau by strychnine. The patient, a girl of seventeen years, was seized in January, 1845, with chorea, which seemed to be produced by suppression of the menses. The menses reappeared in the course of six weeks, and the chorea disappeared about the same time under the influence of sulphureous baths. In October, 1845, the menses were suppressed anew. The chorea reappeared, occupying this time both sides, with a predominance in the left; there was marked diminution of sensibility and power of articulation, and manifest enfeeblement of the intellect and memory. The patient entered the Necker hospital on the 22d of December.

M. Trousseau prescribed the syrup of strychnine in doses gradually increased, containing at first the fifth part of a grain of strychnine, which, when it had been augmented to a grain a day, produced a very sensible therapeutic effect. The chronic agitation almost entirely ceased, the intelligence became much more clear, the memory much more active; but the patient still complained of lively itchings of the head, much stiffness of the jaws, without any convulsive twitchings elsewhere. The syrup was continued during some days in the same dose, then the quantity was progressively augmented until the patient took two grains of strychnine in the twenty-four hours. After two months of treatment, the chorea completely disappeared. The administration of the strychnine was not wholly suspended at once, but gradually diminished. The patient quitted the hospital entirely recovered.

The menses had not reappeared, but their suppression was now owing to her conception. The cause which produced the chorea in the first instance was evidently the suppression of the menstrual flux. It is probable that this is one of the most common causes of chorea, though it is seldom that the influence of the menstrual discharge in the production of this disease is so distinct. It is equally important to remark the rapidity with which the recovery was effected. The therapeutical application of strychnine is a difficult medication, requiring great prudence and care; at the same time, it is one of the most powerful remedies of the materia medica. This, however it may be to others, to me is a very interesting case.

I was disappointed in Andral, who lectures on pathology and general therapeutics at l'Ecole de Médicine, as a lecturer. He reads most of his lectures, and does so very rapidly, not as distinctly as some, hardly ever lifts his eyes from the paper, and seldom makes a gesture. Gerdy, one of the surgeons at la Charité and lecturer on pathological surgery at l'Ecole de Médicine, is a pleasant speaker, rapid, distinct, animated, becoming at times very earnest. His voice is not a good one, lacking compass. He is a fine sized man, with large head, thick black hair, which is here and there sprinkled with a few gray hairs, black eyes, and large nose; indeed, all his features are large. His age I should take to be fifty years.

In my last letter I spoke of M. Roux and the ligation of the femoral artery for what he pronounced to be an aneurism of the head of the tibia. The diagnosis of this case has created an unusual amount of interest. The editor of the *Gazette des Hopitaux*

differs from M. Roux, and believes the affection to have been a bloody tumor complicated with cancerous degeneration, instead of simply a *tumeur fongueuse sanguine*, analogous to erecetile tumors of the soft parts, produced by dilatation of the arterial capillaries of the substance of the bone. The difficulty of diagnosis between an osseous aneurism and a cancerous tumor of the bones accompanied by pulsations, is extremely great. The points of resemblance between the two are numerous and striking; both are intimately connected with the subjacent bone; a loss of substance of the bone is distinct in each; in both, an osseous lamella affords crepitation; an expansive pulsation and fluctuation is detected in each; and lancinating pains, which not long ago were considered as pathognomonic of cancerous affections, are experienced alike in osseous aneurisms and other tumors which present no degenerations of a grave description. M. Nélaton, surgeon to l'Hôpital St. Antoine, who has published an admirable volume entitled "Traité de Pathologie," and is on the eve of bringing out a second, gives in substance the following, which may assist in distinguishing the two affections:

1st. Osseous aneurism, existing without any complication, disappears completely or almost entirely under continued pressure; while an encephaloid tumor, although it may be made to sink in some degree under pressure, always presents a mass of cancerous tissue which cannot be dissipated.

2d. Cancerous tumors of the bones which present pulsations are accompanied, ordinarily, by a *bruit de souffle*, which is never heard in osseous aneurisms.

Dr. Marati, of Naples, in a practice of twenty years, has had occasion to treat a great number of patients afflicted with malignant pustule and anthrax. He has never employed the red iron nor any other caustic in cases of this kind, but has had recourse exclusively to extensive frictions with mercurial ointment morning and evening; he has applied thus from a hundred to a hundred and twenty-five *grammes* of the ointment in a day to the red and tumefied surface, and by the aid of this simple remedy alone, has succeeded in all except two cases, upon whom the disease had made too great progress before the friction was commenced, in circumscribing the affection, putting a stop to accidents, favoring the fall of the eschar, and finally in obtaining a rapid recovery.

In the great number of cases submitted to his observation, he has not seen a single one in which salivation was produced by this energetic hydrargic treatment, and he has administered it indiscriminately to old and young subjects, adults and pregnant women in all stages of the disease.

This remedy is rendered even more precious from the fact of the practitioner's being able to associate it with cauterization and the internal use of the spiritus mindereri, or of the ammonia itself, in doses appropriate to the condition of the patients.

M. Velpeau delivered, a short time since, a very interesting lecture on inflammation of the submaxillary and sublingual glands. He gave the following as signs which establish a distinction between inflammation of the ganglia and that of the salivary glands:

In the glands, the inflammation commences in the interior of the mouth; the pain is felt in this point: in the ganglions, the contrary is the case. In inflammation of the glands, the salivary function is troubled; the mouth is probably dry at first, then the secretion of saliva is augmented; there is no external inflammation, no redness of the skin, no *empâtement;* the tumor is movable: these signs are never present in ganglioni phlegmasia. In the latter (ganglioni phlegmasia), suppuration is very easy: in the glands, resolution is most frequently the result; suppuration occurs with difficulty, and inflammation gains but a small portion of the neighboring tissues: the opposite is witnessed in inflammation of the ganglia, eight days hardly elapsing without the formation of pus. Finally, when the salivary glands are the seat of a phlegmasia, the tongue is pushed against the palate, and deglutition is perhaps embarrassed: when it is the ganglions which are affected, the patients may complain of pain in swallowing, but there is no mechanical obstacle.

The treatment of these two affections, although alike in some respects, differs in many points. In inflammation of the ganglions, venesection is generally useless, while leeches produce a good effect; then after some days comes the surgical treatment, afterwards cataplasms and vesicatories. In inflammation of the salivary glands, bleeding from the arm is more efficacious than topical bleedings; afterwards a tolerably deep incision by the mouth stops, ordinarily, the inflammation, and brings about a speedy amelioration.

M. Louis, at the *Hotel Dieu*, had in September last a case of pulmonary consumption, the march of which was rapidly to a fatal termination. The patient, admitted on the 14th, was a woman æt. twenty-eight years, a semstress of delicate constitution, lymphatic temperament, who has borne two children at four years' interval, and enjoyed habitually good health. The first confinement was happy, the second was also terminated easily, but a fortnight after she was attacked with diarrhœa which has continued ever since. Four months have elapsed since the last confinement. The patient dates the beginning of the affection fifteen days after. It made its appearance in the form of a very abundant diarrhœa, mounting as high sometimes as eight or ten stools during the day. Sometimes small quantities of blood were mixed with the fæcal matter, which, save that it was more liquid, resembled the white of an egg. Slight pain in the abdomen, cough which has existed about the same length of time as the diarrhœa, small, however, and until lately dry: some sweats at night; no pain in the breast.

Bronchial respiration at the summit of the right lung, and broncophony. The respiration is rude and a little *soufflante* throughout the whole extent of the lung: sufficiently good in the left. Some pricking in the throat. The voice is unaltered. Interrogated as to the seat of her suffering, she places her hand in the region of the larynx. She complains also of a pricking and uncomfortable heat behind the sternum. This pricking provokes the cough, which, without being exactly painful, is laborious. After fits of coughing the pit of the stomach becomes painful, owing probably to the fatigue caused by the shocks. Since the last twelve days she has expectorated after coughing, without difficulty, a thick, white, viscid matter, a little gray in places, without traces of either blood or pus. No appetite; lively thirst. Food appears bitter to her. The abdomen, a little sensible to pressure, is well formed, presenting no tumor even when strongly pressed. Numerous stools; eight or ten a day. Rice water, syrup of gum, with extract of opium injection, laudanum 10 drops; diet.

16. The stools have been less numerous than the day before yesterday; only one during yesterday. Vomited three times. The general feebleness is very great. She replies slowly and with difficulty; countenance sunken; skin moist; pulse frequent, 104 *a* 108; sensation of weight and heaviness about the head; movements slow, intellect clear; no vertigo; a little drowsiness.

The phenomena furnished by auscultation remain the same: some crepitation at the middle part of the lung. Two pills of the subcarbonate of iron; laudanum injection; diet.

19. Six liquid stools. Since her entry the patient has grown sensibly thinner. Feebleness increased every day: visage greatly altered. The eyes are surrounded by a bluish circle. There is a pale yellow tint of the skin; lips pale; projecting cheek bones. Same treatment.

23. Continuance of the diarrhœa; the tongue is furred in the middle, and red upon the edges. No appetite; lively thirst. Respiration grows more and more laborious every day. Expectoration abundant, characteristic, containing pus, evidently tuberculous, swimming in a mucous liquid. From this moment all the above mentioned symptoms became greatly aggravated. Seven to eight stools a day; pain in the belly; emaciation progresses rapidly; pale yellow tint of the skin; frequent cough; abundant expectoration, characteristic. The eyes are deeply sunken in the orbits. Feebleness extreme. The patient nevertheless says she does not suffer, and complains only of a little oppression and difficulty of respiration. She died on the 5th of October. The autopsy revealed numerous granulations of the left lung, especially at its summit, and a considerable crop of tubercles in the crude state, of very small volume. The right lung contained a large cavern in the summit, which appeared of recent formation. Numerous granulations and tubercles, some in the crude state, others beginning to soften, were observed throughout the entire extent of the organ. The mucous membrane of the large intestines was of a bright red color; no tubercles were found in the mesentery. Other organs healthy. The condition of the large bowels accounts for the obstinate diarrhœa in this case.

Acute phthisis, says Lænnec, is the product of a tuberculous affection of the lungs, which, latent, during a longer or shorter period, unmasks itself suddenly, produces an acute fever, emaciation and generally symptoms so grave, that the patient is carried off perhaps in six weeks, or a month, and sometimes in less time.

Sometimes the march of acute phthisis is so rapid, that it lasts only two or three weeks, and M. Louis has detailed, in his work on pulmonary phthisis, the case of a man who died in twenty days after the first signs of phthisis were observed. The lungs contained caverns.

Whatever may be the opinion adopted, as regards the above described case, in relation to the latent condition of the tubercular deposit, it is unquestionable, that among certain subjects phthisis progresses as an acute disease, especially in the period of softening. It is allowable to suppose, in the case in hand, that the disease existed for some time before it manifested itself. Many authors, we know, state, that when a phthisical woman conceives, the phthisis remains stationary during pregnancy, and assumes a new and greater intensity a short time after her confinement, which seems to become the signal for the revival of the disease. There are other circumstances which hasten the march of phthisis; such are small-pox, measles, scarlatina, and especially bronchitis and pneumonia.

M. Béhier, one of the physicians of *La Charité*, has had during the last few weeks several interesting cases of small-pox in his service. A female, twenty-eight years of age, slept for some nights next to the bed of a patient laboring under variola: one morning, in making up a bed, she touched his person. Twenty days after she was seized with chilliness, followed by heat and a sensation of general lassitude, with pains in the loins, felt particularly in movements of the limbs. She complained of soreness of the throat, with tolerably intense oppression; the tongue was white, the eyes weeping, and the palate the seat of papulæ. The most remarkable phenomenon in this case, and it is on this account that I have reported it, was the form and volume of the pustules, which were small, pointed, resembling simple acne, and not presenting the appearance of pustules with wide bases which are observed in variola. This modification in the eruption alone gave rise to some fears of unpleasant complications. And this is a character which it is well to bear in mind, that whenever the pustules present themselves in this way, there is reason to anticipate an abnormal march, or some grave epiphenomenon in the disease. The third day a violent delirium declared itself; the patient jumped out of bed and ran across the ward. She complained greatly of her throat: her pulse was at first 74; then 84; then 90. Six leeches were applied behind each ear. Calomel was given internally; two large blisters to the lower extremities, after having previously used sinapisms. The third day after the delirium the pustules became less distinct, and the symptoms improved. This is a remarkable fact—the patient had been vaccinated, and should have had varioloid; but, on the contrary, her symptoms were exceedingly grave. It is generally the case, when a patient who has been vaccinated is attacked with variola, that the disease is more mild than if vaccination had not been performed. An opinion prevails very extensively among physicians, that in variola the pustule is umbilical, or pointed, and that it is not so in varioloid. This is proved to be altogether erroneous. In variola, umbilical pustules are but rarely seen, and the only real difference between it and varioloid is in the time of desiccation. In the former, between the eruption and the desiccation, there is a distinct period, while in varioloid the pustules appear, become dry, and the scales are formed and fall off very rapidly.

There should be no importance attached to the umbilical shape of the pustules in these diseases, according to M. Béhier, who has made extensive and careful researches on the subject, both at *l'Hopital Saint Louis*, and *l'Hopital des Enfans*, where he has had numeruus opportunities of observing these diseases. As a general rule, variola does not offer umbilical pustules. The history of this patient, after her recovery from variola, shows the possibility of complications posterior to the principal affection. The parotid of the left side of her neck inflamed, and the abscess was opened. Erysipelas seized first this side, and afterwards the other, and was followed by anasarca, which commenced in the face, obstinate diarrhœa, and albuminous urine. The same remedy was employed in this condition, as in a similar case supervening upon an attack of scarlatina: vapor baths produced sensible amelioration of the symptoms.

In the treatment of hydrocele, M. Blandin does not believe that the nature of the liquid injected into the vaginal tunic exercises any sensible influence upon the results of the operation. He employs, with equal confidence, the vinous decoction of roses de Provins, tincture of iodine, etc. It is only necessary that the liquid should possess irritating properties, in a sufficient degree to provoke an adhesive inflammation, in order that its use may be followed by success. He prefers a simple mixture of three parts of water to one of rectified alcohol. This liquid possesses the advantage of being always at the hand of the surgeon, and not requiring, as the vinous decoction does, a manipulation more or less tedious; and what is of more importance, it possesses over the preparations of iodine the advantage of leaving no stain on the instruments, or on the linen of the patients.

In the *Journal Connaissances Médico-Chirurgicales*, and the *Gazette des Hopiteaux* I see favorable notices of the *monésia.* One writer thus speaks of it: Monésia is easy of administration: it is active even in minute doses, 12 *a* 16 grains per diem being usually sufficient. It may be given in the form of pills of 4 grains each; of syrup which contains 6 grains to the ounce; of alcoholic tincture, which is employed both externally and internally, of the strength of 30 grains to the ounce: finally, it may be used in the form of an ointment, composed of one part of the extract to seven of hogs' lard. These proportions of course may be modified at the discretion of the physician.

We have noted, continues this writer, that in all cases where astringents are indicated *monésia* is a precious remedy; like catechu it is a powerful astringent and at the same time stomachic; it neither irritates the stomach nor dries the mouth. The preparations of monésia may be regarded as specifics in passive hemorrhages, diarrhœas and chronic catarrhs, and in many diseases where there is feebleness and softening of the organs or tissues. Our successes have constantly verified these opinions. We see with regret, that a substance so efficacious and yet so incontestably harmless is not oftener used in therapeutics. It has rendered service in that intestinal phlegmasia, designated cholerine, which prevailed with so much severity in London, and which is perhaps as frequent though less violent, at Paris. In fact, against the fluxes of the belly, these serous diarrhœas sometimes sanguinolent, monésia is an excellent remedy that we cannot too much recommend to our brother practitioners.

Recently we have obtained by its use the cessation of a hematemesis which had resisted the usually employed remedies. We conclude by citing some cases.

The first of these was an affection bearing a close resemblance to the *noli me tangere*, and consisted of a button situated upon the cheek: a great number of topical applications had been used without result; cauterizations of various kinds had only brought the little tumor to a state of ulceration without producing cicatrization: we were disposed to resort to the arsenical paste when we were struck with the idea of sprinkling this ulcerated surface with the powdered extract of monésia: prompt and complete success was the result, and for two months the recovery has been maintained. The other observation, not less remarkable, relates to an excoriation of rather a carcinomatous character upon the end of the nose—an excoriation which had existed three years, and which began to extend itself notwithstanding the well-advised treatment which had been practised; cauterization had been resorted to without avail. We prescribed lotions, frequently repeated, with water containing 60 grammes of tincture of monésia to the pint; and we have obtained a complete and durable recovery.

The present number of hospitals or almshouses in France is 1338, having a revenue of 53,632,992 fr. There are 39 institutions for the education of the deaf, dumb and blind youth of the kingdom, in which at present 1675 pupils are receiving instruction. The number of deaf and dumb persons in France is computed at 20,000 to 25,000, and the number of blind at 12,000 to 15,000. As to the *enfants trouvés*, the number cannot be accurately made out, but 123,394 have been received at the Foundling hospitals, of which there are 144, in a single year. The number of indigent lunatics is 12,286, who are supported by the government at an annual expense of 4,326,138 francs, and by the humane and judicious treatment a large number of these unhappy beings are every year restored to reason, and to their families and society.

In a late English paper I observed an analysis of gun-cotton made by Professor Graham, of London. Exploded in a glass tube so as to collect the gaseous products, 53.33 grains of the cotton yielded 100 cubic inches of gas, of which the composition was as follows:

| | | |
|---|---|---|
| Carbonic acid | 14.284, | or 2 volumes; |
| Cyanogen | 7.143, | or 1 volume ; |
| Nitric oxide | 35.715, | or 5 volumes; |
| Carbonic oxide | 35.715, | or 5 volumes; |
| Nitrogen | 7.143, | or 1 volume. |

Besides these aëriform bodies, a portion of oxalic acid is precipitated, and a considerable quantity of water results from the combustion.

Near the *Marché neuf* stands a plain Doric building of one story, divided into several rooms, and a large entry or hall. Here, in a little chamber separated from the entry by windows running from the ceiling almost to the floor, are deposited for the space of three days the bodies of those unknown and unfortunate beings who have committed suicide, or met with death by accident. This room contains ten tables, or inclined planes, upon which the dead are laid: over their heads, upon pegs which are driven around the wall, are hung their clothes; and these are allowed to remain long after the bodies have been removed, in order still further to promote the recognition of their owners. After the lapse of three days, unless the bodies are claimed, they are removed and buried at the public expense.

The same feeling of unnatural curiosity which prompts hundreds of people to flock about the scaffold to witness the dying struggles of a fellow-creature, keeps constantly in and around the *Morgue* numbers of men, women and children, ever on the alert to see the cart drive up which contains the remains of some unhappy Frenchman, who, tired of the fierce struggle of life, has shuffled off his mortal coil, by a somerset from a window in the fifth story, or a dive into the Seine from one of the many bridges which span it. Or, perhaps it may be the body of a poor girl, who listened to the voice of deceitful love, was seduced, became a mother, and then, to quiet the stings of conscience, or appease the stern calls of hunger, swallowed a few grains of arsenic, or a few drops of prussic acid, Or, as the other day when I passed it, the "green-eyed monster" may have gained the ear of some tricky husband, and prompted him to kill and quarter the supposed, perchance the real, author of his dishonor.

The crowd is a motley one, and, of course, has its Billy Sniffle. The mender of soles crosses over from his shop, which is not far off. As he enters the door he ties the wax-ends about his neck that they may not be lost while he elbows his way towards the tables. After having feasted his eyes to his heart's content, he turns and addresses himself to the woman by his side, who, as is usual in such cases, is acquainted with all the circumstances of the catastrophe. She begins her narrative, and soon becomes a centre about which a group collects and listens, as she oracularly delivers what she has heard "that somebody told some one else." The market woman passes by; she cannot resist the temptation, but, looking about for a convenient place where she may deposit her large and heavily loaded basket, soon has thrusted her nicely handkerchiefed head into the assembly, where she drinks in every detail. Thus filled, she takes a long look at the bodies, returns, asks some one to assist her to shoulder her basket, moves on, and, in turn, becomes herself a magnet, around which curious women and wondering children gather to listen to her narrative, and at last go away to repeat the sad, eventful story.

Many garments, of males and females, hang on pegs around the room. They are of all descriptions—here a gown, there a coat; here a hat, there a bonnet; on this peg a pair of coarse, heavy boots, on that kid slippers; here a cravat, there a shawl; now a cloak, and by its side a chemise; here a pair of fine black trowsers, and opposite to them the patched and greasy breeches of the laboring man, who, perhaps sick of his destiny, unknown, friendless, and in want, loosed the silver cord by blowing his brains out with powder and lead that he had purchased with his last *sous.*

The bodies of about three hundred persons are annually exposed at the Morgue, of which five-sixths are males.

Having spoken of Morgue, the *dead house* of Paris, I will conclude by a brief reference to the final resting place of the living of this great metropolis. *Pere la Chaise*, the chief cemetery of Paris, was at one time the country seat of the Jesuits. It was consecrated as a burying ground in 1804; it lies on the north-east of the city, on the side of a hill, and contains one hundred acres, surrounded by a wall. Besides the many who go there to hang garlands on the graves of their friends and relatives, the number and beauty of the tombs, the extensive panorama which is caught from the hill top, and that undefined interest and curiosity which prompts one to visit the resting place of the dead, contribute, on every beautiful day, to assemble hundreds of people within the enclosure; and, according to my taste, there are few places about Paris which so well repay the time and trouble of a visit.

The second day of November is *le jour des morts*, (all-souls-day,) the ceremonies of which interest a stranger, and especially one from a protestant country. For several years the day had been unfavorable; but a more glorious one than the last could not have been desired, save that the ground was rather damp, and, as was anticipated, more persons were present than had been for many an all-souls-day before. The cemetery, divided by numerous roads and paths, was the scene of many phases of human condition and character during the day. From morning till evening it was crowded with people, all in their fête day clothes. With the three periods of morning, noon and evening, changed the character of the visitors. Early in the day, the poorer people, those who, notwithstanding that it was a holiday, were not able to spend it as such, came, sought out the simple and unadorned graves of those they had buried, and, hanging their single garlands upon one of the wooden rails which marked the spot, turned away with a grief as profound perhaps as those who, at a later period, descended from their carriages, and amid the rustle of silks strewed fragrant flowers and costly garlands upon marble tombs, and again seated themselves and were whirled away to scenes where the dead are forgotten.

In the streets leading to *Pere la Chaise* on the second of November, exposed from every door near the cemetery, are garlands for sale, made of *l'Immortelle*, a flower which is dried and retains for a long time its color. They are either perfectly plain, or have on them, "To a dear brother," "To a kind mother" &c. &c. They are laid upon the tombs, around or in them, or placed over them on a rod protected by a cover: some were withered and dead, others were fading, others had been removed and were trampled under foot, whilst others again were fresh. One might have been disposed to speculate on the different stages of grief suggested by the fresh and dead garlands, but that, on returning from the gate of the cemetery, he remarked hundreds of those whom he had seen bring in and deposit the flowers, standing and enjoying the the feats of a juggler. While I was in the cemetery a burial took place, and as the funeral car rolled along its solemn way no head which it passed remained covered. Hundreds stood by to see the coffin lowered, and as it was covered out of sight the springs of grief in many a heart were made to flow afresh. When a funeral takes place here, as it moves down the street, every one removes his hat and remains uncovered until it has passed. There are many trees in the cemetery; I was there in the season of "the yellow leaf;" and as they fell and were trodden upon, one felt that before the fall of next year's leaves hundreds of those human beings who walked then carelessly among them, would themselves be entombed and as utterly forgotten. The man gives a *sous* for a garland by which to testify his annual remembrance of his brother, deposits it upon the grave, turns away, saunters through the cemetery, goes out, is attracted by the loud spoken advertisement of a juggler, pays him a *sous*, and moves on, to return another year, renew the garland and patronize a mountebank! There are upwards of 15,000 tombs in this cemetery, upon which it has been estimated that nearly twenty millions of dollars have been spent. They are very slight and will not last long, a circumstance to be regretted. There are crosses and virgins in great numbers of these, and very many contained burning wax tapers. The monuments represent pyramids, sepulchral chapels, temples, obelisks, columns, urns, mausoleums, etc.— The tomb of Laplace is of white marble, surmounted by an obelisk, which is capped by an urn, ornamented with palm branches and a star encircled by inscriptions alluding to his works. A cenotaph, crowned by a fox, in black marble, and ornamented by two bas-reliefs in bronze, one representing the fable of the *wolf and the stork*, and the other the *wolf and the lamb*, you will recognize as the the tomb of La Fontaine.— One other and I have done: the purport of it is this—"To the memory of —." This monument was erected by his "disconsolate widow." She informs her friends and the public generally, that "she still continues the business at the former stand, rue —, No. —." Of course, all who read this inscription are curious to see its careful author, and many thereby, no doubt, are induced to "open an account" with the "disconsolate" creature.

Paris, December 30th, 1846.

